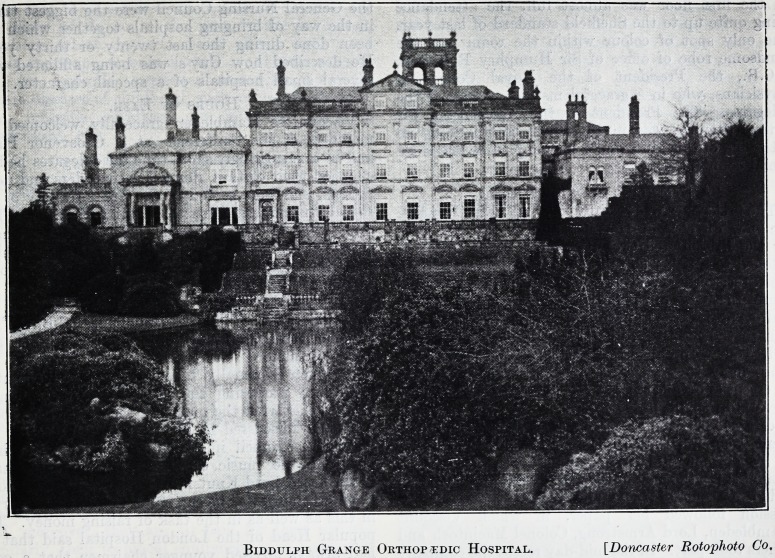# Opening of Biddulph Grange

**Published:** 1924-07

**Authors:** 


Biddulph Grange Orthopædic Hospital. [*Doncaster Rotophoto Co.*

### OPENING OF BIDDULPH GRANGE.

The opening by the Prince of Wales of Biddulph Grange as an orthopædic hospital for crippled children marks an important addition to the institutions of North Staffordshire. The gift of Mr. Robert Heath, nearly £30,000 has been spent on the conversion of the house. Biddulph Grange is a large and elaborate modern house in what used to be called the Italian style. The place is, however, chiefly remarkable for the famous gardens created some eighty years ago out of a swampy moor by the late Mr. James Bateman, the pioneer of cool orchid cultivation. Nothing more perfect of the kind has ever existed in England. They are exceedingly varied, with picturesque natural features artfully dealt with, and a plentiful dash of the purely artificial. There are hills and dales, cascades and turrets, a lake and great masses of rock. There are Chinese and Egyptian gardens and an arboretum paved with stones from the Appian Way.

## Figures and Tables

**Figure f1:**